# Concurrent Validation of a Multi-Camera Markerless Motion Capture System Against Inertial Sensors for Upper- and Lower-Limb Joint Kinematics

**DOI:** 10.3390/s26175492

**Published:** 2026-08-29

**Authors:** Carlalberto Francia, Lucia Donno, Gaia Strada, Veronica Cimolin, Mario Covarrubias Rodriguez, Manuela Galli

**Affiliations:** 1 Department of Electronics, Information and Bioengineering, Politecnico di Milano, 20133 Milan, MI, Italy; carlalberto.francia@polimi.it (C.F.); lucia.donno@polimi.it (L.D.); gaia.strada@mail.polimi.it (G.S.); veronica.cimolin@polimi.it (V.C.); 2 E4Sport-Human Peformance Lab, Polo Territoriale di Lecco, Politecnico di Milano, Via G. Previati 1/c, 23900 Lecco, LC, Italy; 3 IRCCS Istituto Auxologico Italiano, San Giuseppe Hospital, Str. L Cadorna, 90, 28824 Oggebbio, VB, Italy; 4 Department of Mechanical Engineering, Politecnico di Milano, Via Privata Giuseppe La Masa 1, 20156 Milan, MI, Italy; mario.covarrubias@polimi.it

**Keywords:** motion analysis, markerless, inertial measurement unit, CapturyStudio, validation, joint angles, rehabilitation

## Abstract

Markerless video-based motion capture is a fast-developing, low-burden and versatile alternative to marker-based stereophotogrammetry, yet independent validation evidence for several commercial solutions is still scarce. This study validates the markerless software CapturyStudio (The Captury GmbH, Saarbrücken, Germany) for the reconstruction of upper- and lower-limb joint kinematics, comparing it with a validated Xsens (Movella, Henderson, NV, USA) inertial measurement unit system. Ten healthy subjects (five females and five males) performed two standardized clinical tasks, a Reach-To-Grasp gesture for the upper limb and a Timed-Up and Go test for the lower limb, recorded simultaneously with eight BTS SMART EVO-DX 2 (BTS Bioengineering S.p.A., Garbagnate Milanese, Italy) cameras operating in markerless mode and a 17-sensor Xsens system. Elbow, shoulder, knee and hip flexion–extension angles were reconstructed from three-dimensional anatomical keypoints provided by CapturyStudio and compared with the Xsens angles through root mean square error in absolute and percentage terms, range-of-motion accuracy, intraclass correlation coefficient (ICC), Spearman’s coefficient (ρ), Bland–Altman analysis and non-parametric Wilcoxon Rank-Sum tests. CapturyStudio reproduced the temporal pattern of all four angles faithfully (ICC ≥ 0.87; ρ ≥ 0.89), with median discrepancies of about 12° for the elbow and below 8° for shoulder, knee and hip and with the best agreement for the upper limb; the main weakness was a systematic overestimation of hip range of motion. Since both systems are indirect measurement techniques, these values represent the discrepancy between two methods and an upper bound on the error of the markerless system rather than its absolute accuracy. Their magnitude is comparable to the changes regarded as clinically meaningful in goniometric assessment, so the system is presently suited to the analysis of movement patterns rather than to the measurement of absolute joint angles. The study is to be read as a technical comparison of two measurement systems, delimiting the conditions under which future clinical, rehabilitation and sports applications may be pursued.

## 1. Introduction

Human motion analysis is the systematic study of body movement with instrumentation for measuring kinematics, dynamics and muscular activity [1]. Kinematics describes the motion of body segments, positions, angles, velocities and accelerations, without reference to the forces that cause it, and plays a central role in biomechanics, sports science, injury prevention and rehabilitation [2]. In the clinical setting, the quantitative description of movement is a crucial tool for assessing a patient’s condition and monitoring progress throughout a rehabilitation program [3]. Marker-based stereophotogrammetry is currently the gold standard for capturing human motion [4,5]. Despite its accuracy, the need for dedicated laboratories, lengthy subject preparation and physical markers on the body limits its applicability in many sports and rehabilitation contexts [6]. To overcome these constraints, inertial measurement units (IMUs), combining accelerometer, gyroscope and magnetometer data through sensor-fusion algorithms, have emerged to estimate segment orientation, offering portability and use in uncontrolled environments [7]; they have shown high reliability relative to optoelectronic systems, although accuracy depends on the task and the plane of motion, with greater variability outside the sagittal plane [8,9,10,11].

Video-based markerless systems reconstruct body movement directly from video, without any sensor on the subject, exploiting computer-vision algorithms to localize anatomical keypoints [12,13]. With respect to marker-based optoelectronic system and IMUs, they are less invasive, cheaper, faster to set up, and allow for acquisitions in a wider range of environments, but their accuracy can be affected by lighting conditions, camera placement, clothing and body occlusions [4]. Human pose estimation (HPE) aims to reconstruct the articulated 3D joint locations of the human body from images or video. Existing methods can first be broadly categorized into single-person and multi-person pose estimation approaches [14]. From a methodological perspective, pose estimation methods include 2D approaches, typically detection-based or regression-based [15], as well as 3D approaches, which, particularly for multi-person settings, can be divided into two-stage methods that lift 2D poses to 3D and direct one-stage 3D estimation methods [16]. Despite remarkable progress in 2D single-person estimation, multi-person and 3D estimation remain challenging, partly because of the scarcity of large, real-world annotated 3D datasets, which limits the generalization of models trained in controlled laboratory conditions [17].

From a system-design perspective, a markerless pipeline is generally composed of the camera system, the body model, the image features and the algorithms used to fit the model [18]. Camera systems may generate a depth map; depth-sensing solutions (stereo or RGB-D devices based on structured light or time of flight) help mitigate shadows, lighting changes and complex backgrounds. Finally, pose-reconstruction algorithms are either generative, fitting a body model to the image data by optimizing a similarity function, which is accurate but sensitive to initialization and occlusions, or discriminative, mapping image features directly to pose, which is faster and more robust [19]. A notable generative example is the Sum-of-Gaussians (SoG) body model, which attaches 3D Gaussian components to a kinematic skeleton and optimizes its pose by maximizing model-to-image similarity, without relying on extracted silhouettes or training data [20]. Several systems have been benchmarked against more historically accurate references, including Microsoft Kinect/Azure Kinect [21] and the BTS SMART-D system [22], with accuracy that is generally better in the sagittal plane and joint-dependent.

These technologies are increasingly relevant in rehabilitation, which aims to restore movement and functional ability after injury, illness or surgery, and where the range of motion (ROM) is a key parameter for assessing joint flexibility and monitoring progress [23]. Because markerless systems enable motion tracking outside the laboratory, they are particularly attractive for tele-rehabilitation and especially valuable for chronic patients or for those living in remote areas with limited access to specialized personnel. RGB-D cameras and IMU sensors are already the most widely used technologies for home-based rehabilitation monitoring, with encouraging results in stroke populations, and the possible integration of markerless tracking with virtual reality offers further opportunities for motivating, gamified feedback [24]. Within this framework, a video-based system that can run on inexpensive, widely available cameras would substantially lower the barriers, physical, economic and geographical, to continuous functional assessment.

Among video-based tools, the markerless software CapturyStudio is a promising solution for clinical, sports and tele-rehabilitation use, also owing to its possible integration with virtual reality and to its ability to work with heterogeneous, even consumer-grade, cameras in cluttered or outdoor environments. However, independent validation studies are very limited, which is currently the main obstacle to its broader adoption. Harsted et al. [25] provided the most relevant evidence, reporting, against a Vicon system, RMSEs ranging from 4.3° to 16.9° and intraclass correlation coefficients between up to 0.95 depending on the joint and the plane considered, with the highest variability outside the sagittal plane. Such heterogeneous results underline the need for further investigation.

Beyond confirming or refuting previously reported error magnitudes, the present study addresses three specific gaps. First, CapturyStudio has, to date, been evaluated in a single independent study, conducted on pre-school children and restricted to lower-limb kinematics during jump tasks [25]; its behavior in adults and on the upper limb is therefore entirely undocumented. Second, markerless validation studies almost invariably address either the upper or the lower limb, so the relative performance of one pipeline across body regions cannot be inferred by pooling separate studies that differ in hardware, protocol and reference system; here, both regions are evaluated in the same participants, with the same camera set-up, the same comparison system and the same processing chain, which permits a direct distal–proximal and upper–lower limb comparison not otherwise available. Third, the system is deliberately evaluated under two unfavorable conditions: against an inertial rather than an optoelectronic comparison system and with grayscale infrared cameras rather than the RGB hardware for which the software is optimized. The contribution of this work is therefore empirical and consists in delimiting the conditions under which a commercial markerless pipeline can and cannot be relied upon, which is a necessary precondition for any downstream clinical or rehabilitative deployment.

The aim of this work is therefore to validate CapturyStudio for the reconstruction of upper- and lower-limb flexion–extension kinematics, comparing the joint angles reconstructed from its 3D keypoints with those estimated by a validated Xsens IMU system. Both selected gestures are clinically relevant in human motion evaluation and are among the most widely used in motor rehabilitation and assessment: the Reach-To-Grasp for the upper limb [26,27] and the Timed-Up and Go for the lower limb [28,29].

## 2. Materials and Methods

### 2.1. Motion Capture Systems

An inertial system was chosen as the comparison system instead of an optoelectronic one for three reasons. First, the BTS SMART EVO-DX 2 (BTS Bioengineering S.p.A., Garbagnate Milanese, Milan, Italy) cameras used here cannot operate in marker-based and markerless mode simultaneously, so a stereophotogrammetric reference acquired with the same hardware was technically impossible and would have required a second, independent optoelectronic system operating within the same capture volume. Second, retro-reflective markers and the associated skin preparation would have altered the visual appearance of the subject in the very video frames from which the markerless keypoints are extracted, potentially biasing the tracking that this study aims to evaluate; an inertial reference is visually far less intrusive in this respect. Third, IMU systems have repeatedly demonstrated high concurrent validity against stereophotogrammetry in the sagittal plane [5,7,11], and they represent the reference that is realistically deployable in the non-laboratory settings in which markerless systems are intended to operate, which makes the comparison directly informative for the intended deployment scenarios. It must nevertheless be stated from the outset that the inertial system is itself an indirect measurement technique carrying its own uncertainty, of the order of 1–5° with respect to stereophotogrammetry depending on task complexity. The agreement metrics reported in this study therefore express the discrepancy between two non-gold-standard methods and constitute a combined, upper-bound estimate of the error of the markerless system rather than an estimate of its absolute accuracy.

**Figure 1:**
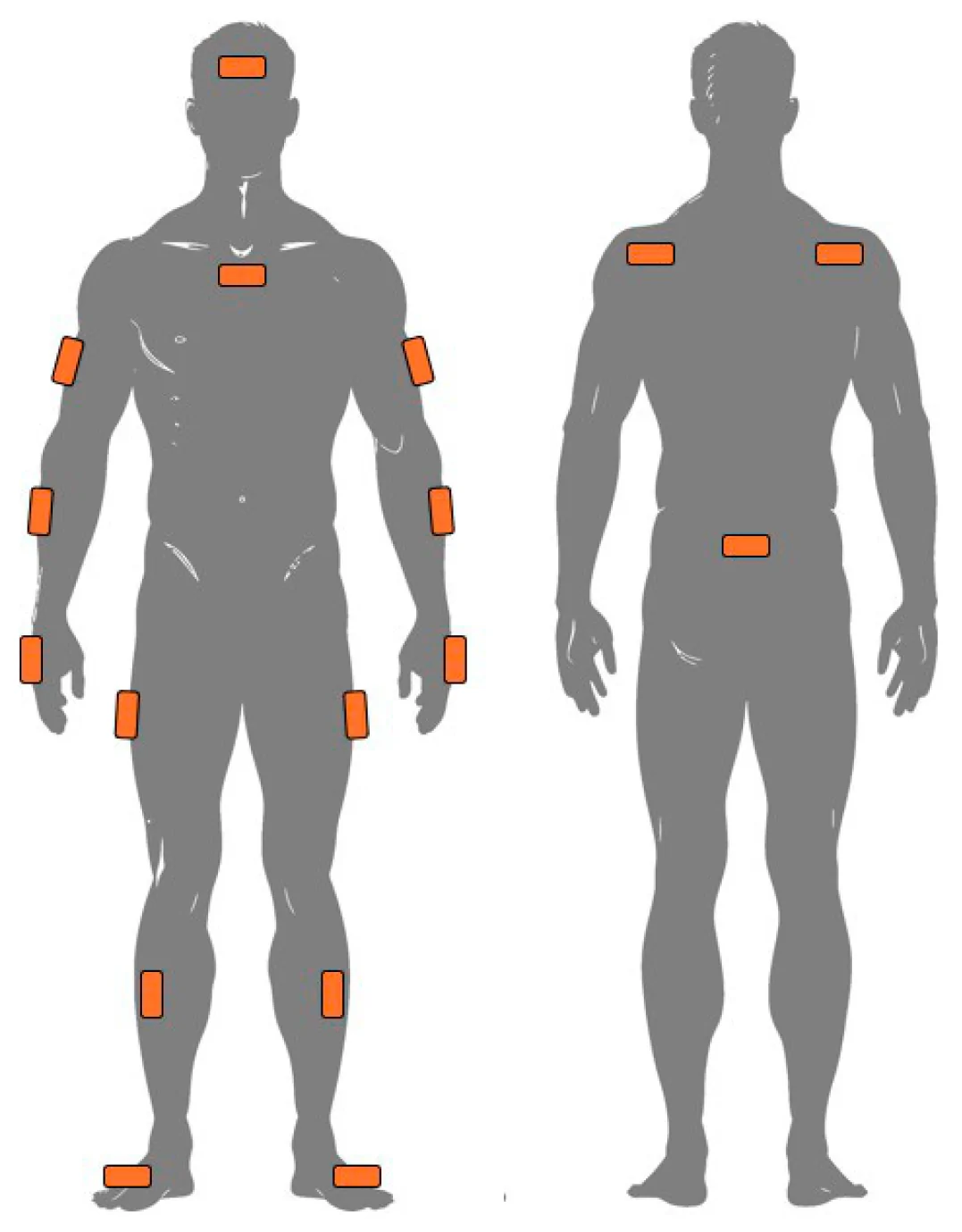
Placement of the 17 Xsens IMU sensors on the subject’s body.

The comparison system was the Xsens MTw Awinda (Movella, Henderson, NV, USA), a wireless inertial motion tracker employing 17 IMUs placed on the head, shoulders, sternum, upper arms, forearms, hands, pelvis, upper and lower legs and feet (Figure 1). Each IMU integrates a 3D accelerometer, gyroscope and magnetometer; Xsens MVN software fuses these signals with a biomechanical body model and outputs joint angles, here exported according to the ZXY Euler convention at 60 Hz.

The system was operated through MVN Analyze (version 2024.2.0, Movella, Henderson, NV, USA). Before each acquisition session, sensor-to-segment calibration was performed using the standard N-pose and walking procedure, and only calibrations rated as good by the software were accepted; otherwise, the procedure was repeated. Acquisitions were carried out in a laboratory free from large ferromagnetic structures and moving metallic equipment, and the magnetic-field quality indicator provided by MVN Analyze was monitored throughout each trial to exclude magnetic disturbance. The MVN engine applies its own sensor-fusion and biomechanical filtering to the orientation estimates, so joint angles are exported already smoothed.

The manufacturer reports a dynamic orientation accuracy of 0.75° RMS in roll/pitch and 1.5° RMS in heading for the individual MTw Awinda sensor. These figures, however, refer to sensor orientation and not to the joint angles derived from it, which additionally depend on sensor-to-segment placement, calibration quality and on the underlying biomechanical model. When validated against optoelectronic references, the Xsens MVN system has shown joint-angle RMSE of approximately 1.2° for short, simple movements and up to 2.8° for longer and more complex tasks, with technical errors reaching about 5° in some conditions and with the dominant source of disagreement attributable to differences in coordinate-system and biomechanical-model definitions rather than to sensor noise [7,8,11]. Agreement is furthermore markedly plane-dependent, being high for flexion–extension and appreciably poorer in the frontal and transverse planes [8,9,10,11]. Accordingly, the Xsens system is used in this work as a comparison system and not as an absolute reference: all metrics reported hereafter quantify the discrepancy between two measurement methods and must not be interpreted as the absolute error of CapturyStudio.

**Figure 2:**
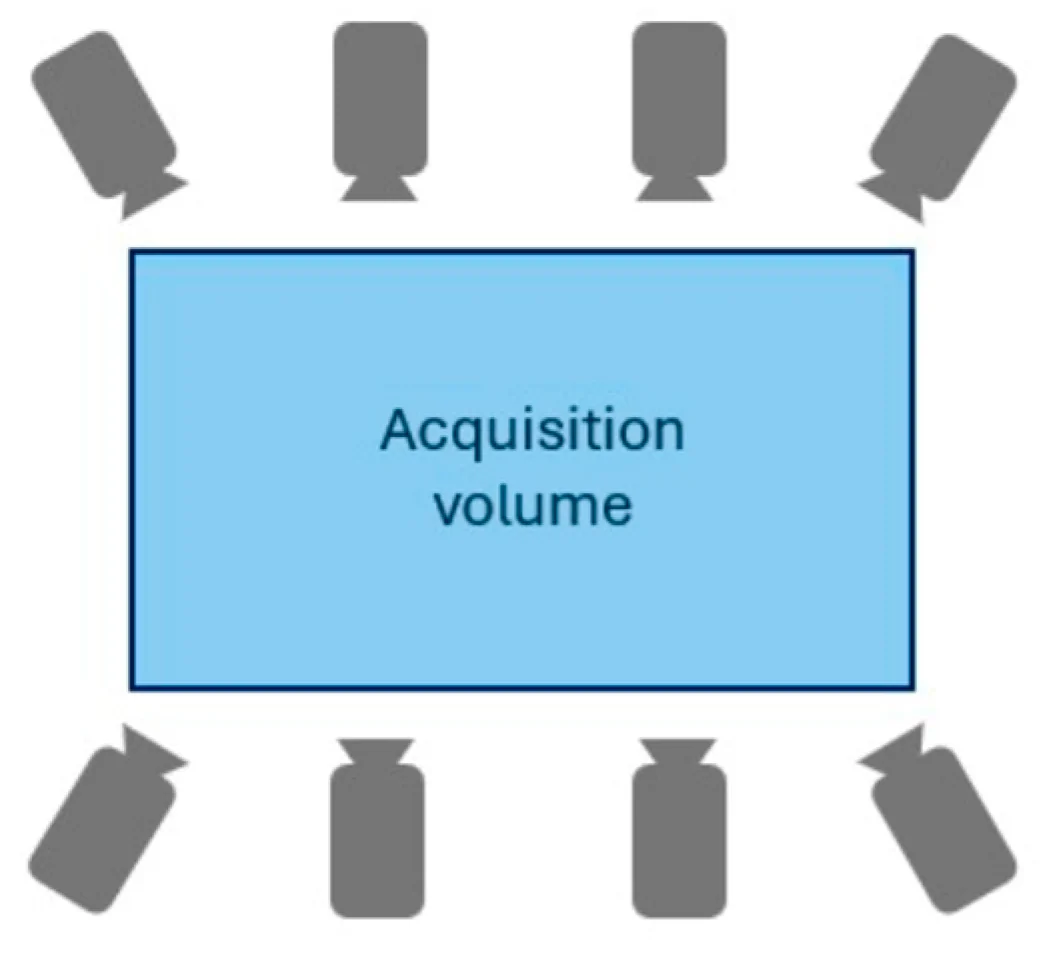
Representation of the acquisition volume and the positioning of the eight BTS SMART EVO-DX 2 cameras around it.

Videos were acquired with eight BTS SMART EVO-DX 2 infrared cameras positioned around the acquisition volume (Figure 2). These cameras support both marker-based and markerless acquisition (not simultaneously); in this study, they operated in markerless mode at 50 fps with a 300 µs shutter, exporting .avi videos and an .xml calibration file.

The videos were processed in CapturyStudio, which fits an actor-specific body model combining a 58-joint kinematic skeleton with a 63-component 3D Sum-of-Gaussians representation (SoG) [20]. Input images are converted into 2D SoG representations via a quad-tree structure; the 3D model is then projected and its pose optimized by gradient ascent on an energy function balancing model-to-image similarity, motion smoothness and joint constraints. The tracking output is a .csv file with the 3D coordinates of the anatomical keypoints for every frame.

### 2.2. Participants and Protocol

This study involved 10 healthy subjects (5F, 5M; age 25 ± 2 years; height 171.60 ± 5.30 cm; weight 63.35 ± 6.80 kg). Inclusion criteria were age > 18 years and absence of neurological or musculoskeletal impairments. All participants read and signed a written informed consent form providing information on the purpose and course of the study.

This research study was carried out in compliance with the World Medical Association Declaration of Helsinki and approved by the Ethics Committee of Politecnico di Milano (protocol code 26/2022).

**Figure 3:**
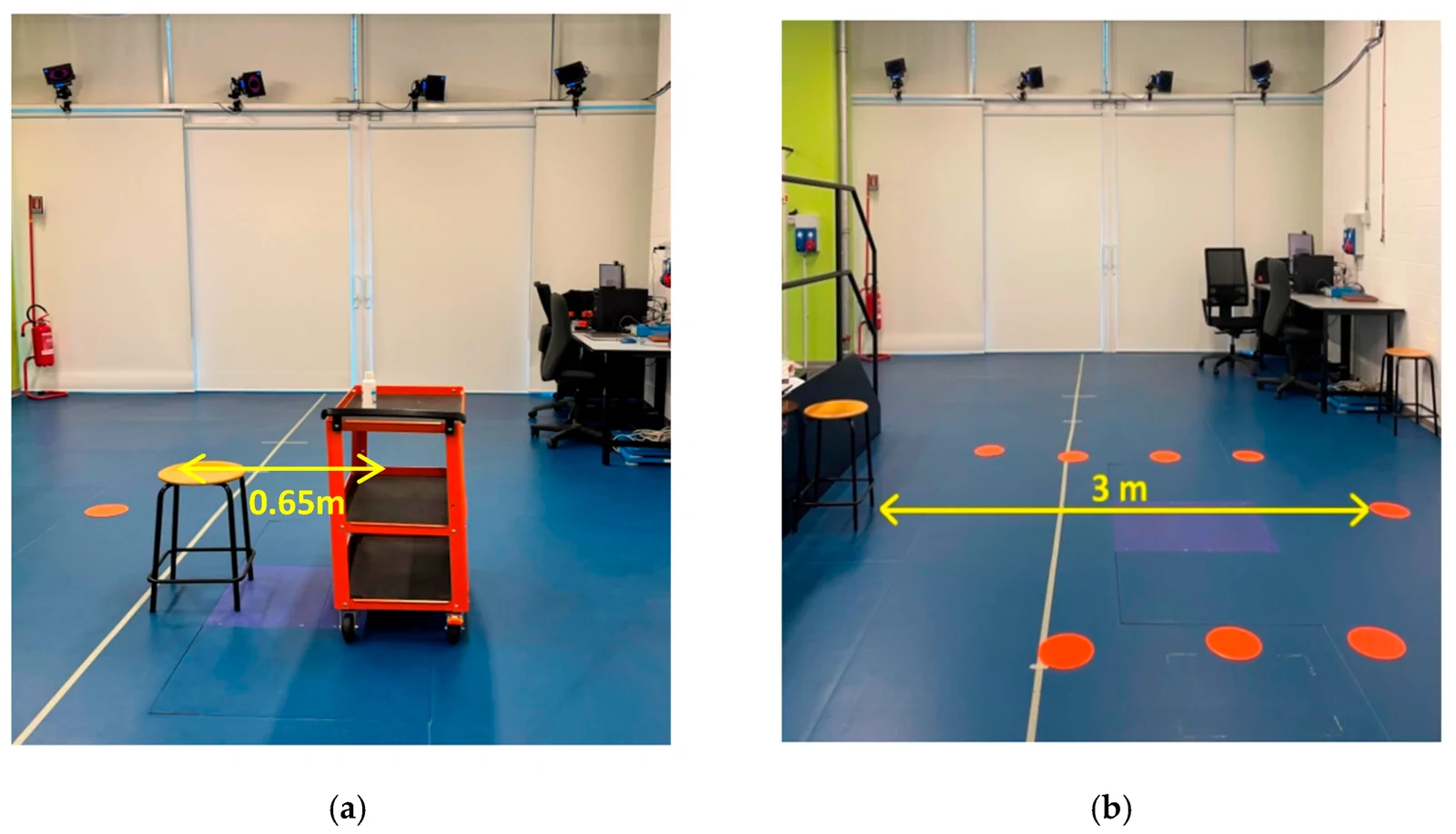
Laboratory setup for the two motor tasks: (**a**) Reach-to-Grasp configuration for the upper limb, with an approximate distance of 65 cm between the stool and the table; (**b**) Timed-Up and Go configuration for the lower limb, with the cone (placed 3 m from the chair) around which the subject turned.

Participants were required to perform two standardized clinical tasks:

**Reach-To-Grasp (RTG):** The seated subject reached, grasped and brought to the chest a small bottle from a table positioned in front of the subject at the distance of 0.65 m and then released it, repeating the gesture five times per arm (Figure 3a).

**Timed Up and Go (TUG)**: The subject stood up from a chair, walked 3 m, turned around a cone and then sat down again. Four gait cycles per limb were isolated from each TUG acquisition (Figure 3b).

Each task was repeated in 10 sessions per subject, for a total of 200 videos.

### 2.3. Joint Angle Reconstruction and Data Processing

**Figure 4:**
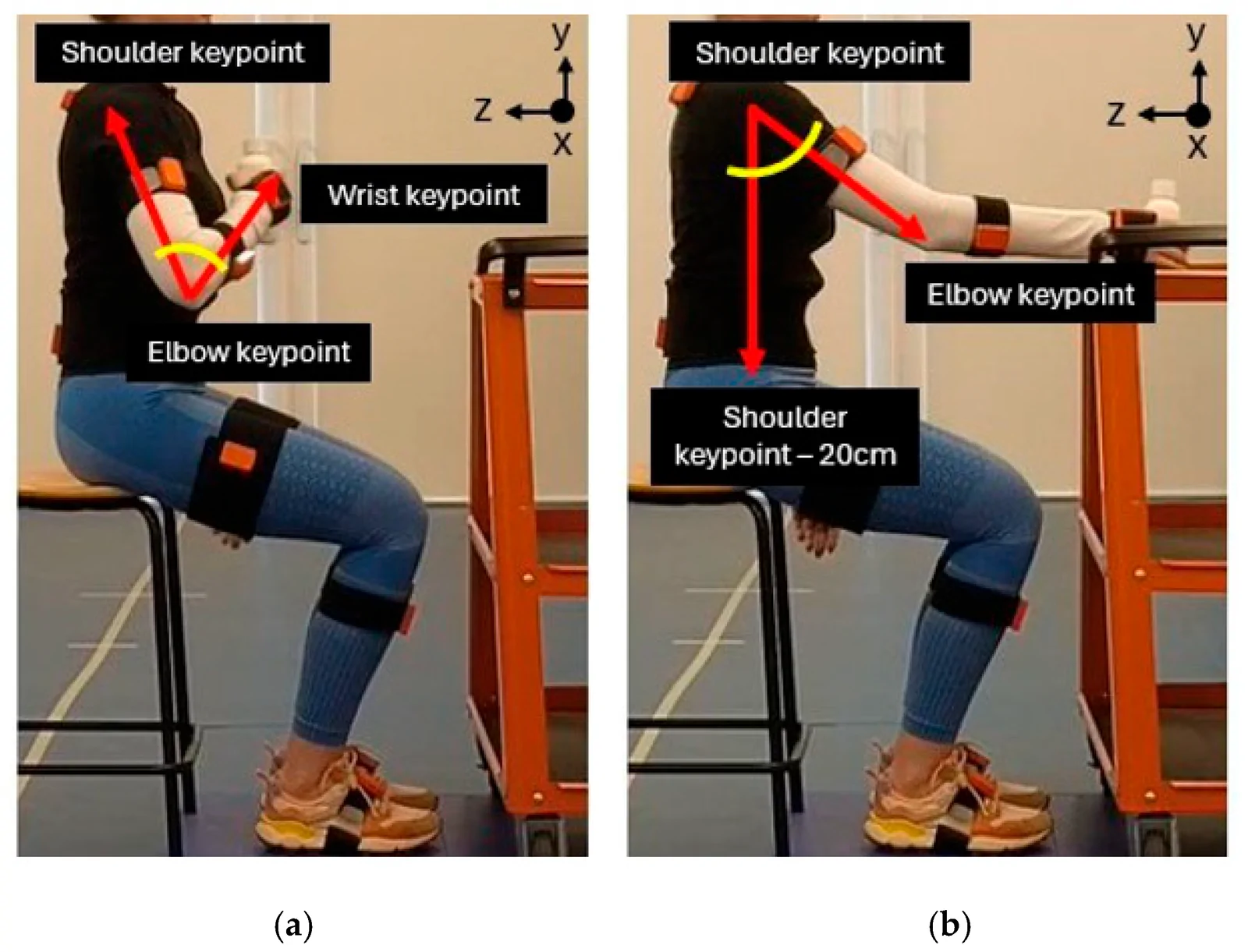
Upper-limb joint-angle reconstruction from CapturyStudio keypoints: elbow flexion–extension (**a**) defined by the forearm and upper-arm vectors; shoulder flexion–extension (**b**) defined between the humerus and a 20 cm vertical reference segment.

**Figure 5:**
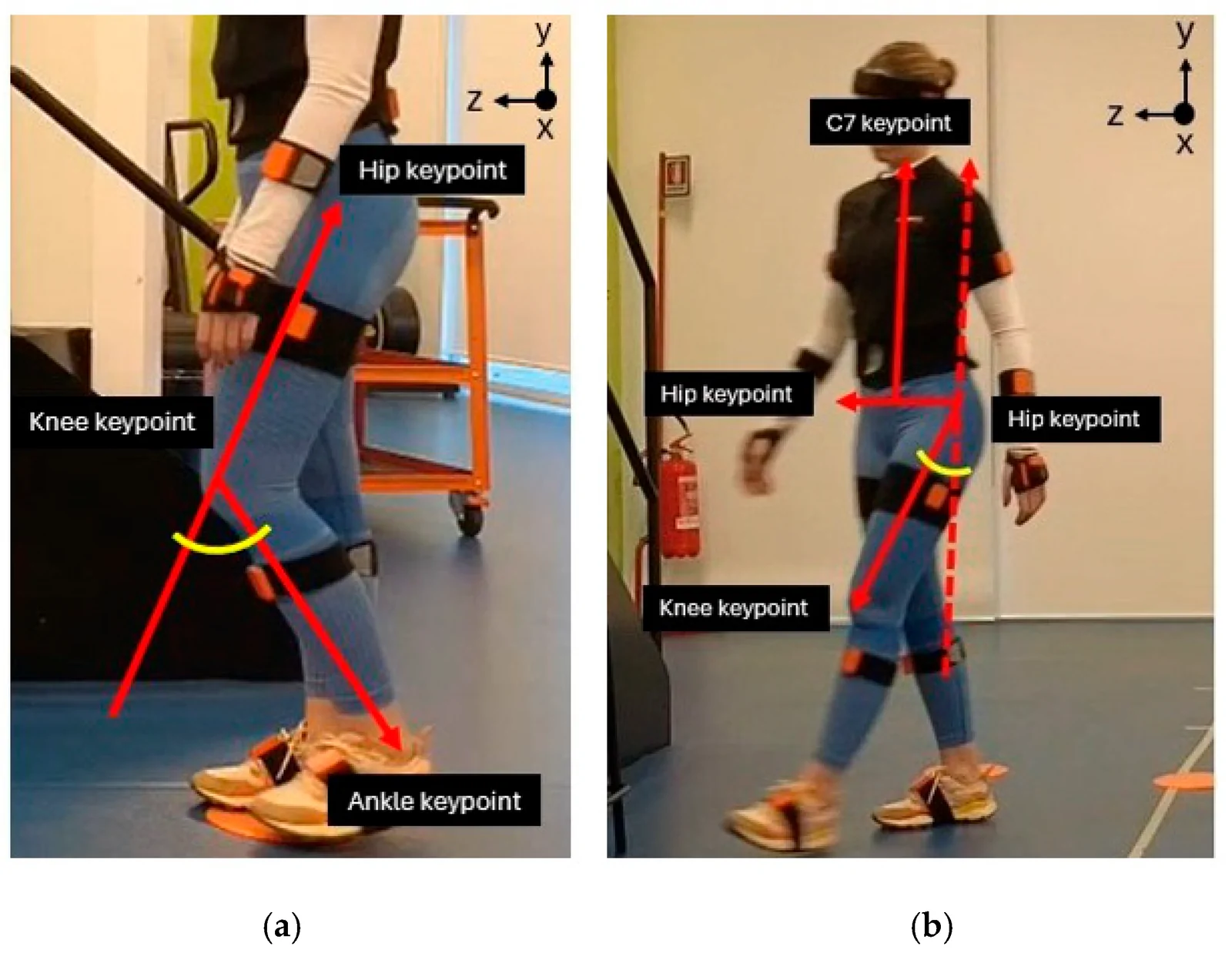
Lower-limb joint-angle reconstruction from CapturyStudio keypoints: knee flexion–extension (**a**) defined by the thigh and calf vectors; hip flexion–extension (**b**) defined between the trunk and thigh segments using the C7 and hip keypoints.

Four flexion–extension angles were analyzed: elbow (EFE), shoulder (SFE), knee (KFE), and hip (HFE). While Xsens provided these angles directly as software outputs, the CapturyStudio angles were reconstructed in MATLAB R2024b (version 24.2, The MathWorks, Inc., Natick, MA, USA) from the keypoint 3D coordinates automatically identified by the software after associating the skeletal model with the subject. For each joint, the proximal and distal segments were defined as vectors connecting the corresponding anatomical landmarks (Figure 4 and Figure 5). Of the 200 recorded videos, 2 were affected by errors in the skeleton association and were therefore excluded, yielding a total of 198 angle datasheets. The joint angle was computed as the angle between the proximal and distal segments according to Equation (1) [30]:

θ = arccos ((v1·v2)/(‖v1‖‖v2‖)(1)

where v1 and v2 are the vectors representing the two body segments. Equation (1) returns an unsigned magnitude, restricted by construction to the range [0°, 180°]. Since the four clinical angles require different neutral references and two of them must distinguish flexion from extension, the construction adopted for each joint is described below.

For the elbow, the upper-arm vector, defined from the elbow to the shoulder keypoint, and the forearm vector, defined from the elbow to the wrist keypoint, originate at the same point so that Equation (1) returns 180° when the arm is fully extended. The clinical EFE angle was therefore obtained as the supplement of the value returned by Equation (1), that is, by subtracting it from 180°, so that 0° corresponds to full extension and increasing values to flexion.

For the knee, the thigh vector, defined from the hip to the knee keypoint, and the shank vector, defined from the knee to the ankle keypoint, are arranged head-to-tail along the limb so that Equation (1) already returns 0° at full extension and increasing values with flexion; the value was therefore used directly, without conversion. For both the elbow and the knee, the joint does not extend beyond its neutral position during the tasks analyzed, so the resulting angle remains non-negative throughout, and no recovery of the sign is required.

For the hip, a local trunk–pelvis reference frame was first reconstructed from three keypoints. The pelvis center was defined as the midpoint between the two hip keypoints; the trunk axis as the vector from this midpoint to the neck keypoint corresponding to the C7 vertebra; and the medio-lateral axis as the vector from the same midpoint to the right hip keypoint, taken on the right side for both limbs so that the resulting frame is identical for the left and the right hip. The antero-posterior axis of the frame was then obtained as the cross-product of the trunk axis and the medio-lateral axis, which, by construction, points anteriorly. The HFE angle was computed as the complement of the angle returned by Equation (1) between the thigh vector, defined from the hip to the knee keypoint, and this antero-posterior axis, that is, by subtracting that angle from 90°. The quantity so obtained is the elevation of the thigh with respect to the frontal plane of the frame, taken as positive anteriorly: in the upright neutral posture, the thigh is orthogonal to the antero-posterior axis, and the angle is 0°, so hip flexion yields positive values and hip extension negative ones over the range from −90° to +90°.

A similar logic was adopted for the shoulder. A vertical reference axis was defined at the shoulder keypoint by translating it 20 cm downward along the vertical direction of the local reference frame, so that the angle returned by Equation (1) between this axis and the upper-arm vector, connecting the shoulder and elbow keypoints, is 0° when the arm lies alongside the trunk and increases with shoulder elevation. Because this expression is unsigned and cannot by itself distinguish flexion from extension, the direction of the rotation was recovered from the antero-posterior position of the elbow keypoint relative to the shoulder keypoint: shoulder flexion, with the elbow anterior to the shoulder, was taken as positive, and shoulder extension, with the elbow posterior to it, as negative. These two constructions are the origin of the negative values for the shoulder and the hip, which correspond to angular excursions posterior to the neutral reference, that is, to extension. The correspondence of the neutral posture and of the positive direction with the Xsens output was verified on the static portion preceding each trial. It should be noted that the four constructions differ in the extent to which they isolate rotation in the sagittal plane. For the elbow and the knee, which are functionally uniaxial, the inter-segment angle is the flexion–extension angle by construction. For the hip, the quantity computed is the arcsine of the component of the thigh vector along the antero-posterior axis of the reference frame; since abduction–adduction is a rotation about that same axis and axial rotation occurs about the longitudinal axis of the thigh, neither alters this component, so the angle obtained is unaffected by the out-of-plane components and corresponds exactly to the first rotation of the ZXY sequence used by Xsens. For the shoulder, by contrast, the angle is referred to a vertical axis and therefore expresses the total elevation of the upper arm, which does not separate flexion from abduction; in the Reach-To-Grasp gesture the movement is almost entirely sagittal, so this component is small, but the shoulder estimate is in principle sensitive to it. These aspects are considered together with the other definitional differences in the following paragraph.

The comparability of the two sets of angles was ensured at the level of the anatomical convention rather than of the computational implementation. Xsens MVN expresses joint rotations as ZXY Euler angles between the anatomical frames of two adjacent segments, as defined by its calibrated biomechanical model, whereas the CapturyStudio angles are computed as signed angles between segment vectors reconstructed from anatomical keypoints. The extent to which the two coincide has been established joint by joint in the preceding paragraph and can be summarized here. For the elbow and the knee, both systems describe the relative orientation of two adjacent long segments, and the definitions are closely equivalent. For the hip, the decomposition adopted here returns the first rotation of the ZXY sequence itself, so the angular definitions coincide, and no approximation is involved. For the shoulder, the reference to a vertical axis makes the estimate an elevation angle, which coincides with the ZXY flexion–extension angle only insofar as the abduction–adduction component is negligible, as it is in the Reach-To-Grasp gesture. All four angles were defined to share the neutral posture and the positive direction of the corresponding Xsens output. What differs between the two systems is therefore not primarily the decomposition but the construction of the reference frames, and this holds to different degrees for the four joints. For the shoulder, the use of a vertical reference segment rather than a thorax-fixed anatomical frame means that any trunk inclination is not removed from the estimate. For the hip, the trunk axis is reconstructed from the C7 keypoint and the hip midpoint and therefore reflects the orientation of the whole trunk, whereas the Xsens model refers the thigh to a pelvis-fixed frame.

A fourth-order Butterworth low-pass filter with a 5 Hz cut-off frequency was applied to the markerless signal to reduce high-frequency noise [31]. No additional filtering was applied to the Xsens signal: MVN software already applies its own sensor-fusion and biomechanical filtering and outputs joint-angle estimates that are intrinsically smooth, with negligible residual spectral content above 5 Hz. Applying a second low-pass stage to an already-filtered signal would have introduced further, and asymmetric, amplitude and phase effects rather than removing them. The filtering step is therefore not asymmetric in its effect: it brings the raw markerless signal to a spectral content comparable to that already produced by the comparison system, and it acts on noise and not on the movement bandwidth, which for both tasks considered lies well below the 5 Hz cut-off.

The Xsens signal was downsampled from 60 Hz to 50 Hz to match the camera frame rate, and the two signals were aligned by selecting the first peak of each signal as the common starting frame, ensuring equal length for frame-by-frame comparison. The residual temporal uncertainty of this procedure is bounded by the sampling interval of the slower system, that is, ±1 frame, corresponding to ±20 ms. Two features of the procedure limit the impact of this uncertainty. First, alignment is performed at a signal extremum, where the first derivative of the angle is null, so that a one-frame error produces a second-order rather than a first-order angular discrepancy at the alignment point. Second, both systems are driven by stable hardware-referenced internal clocks, so no resampling drift accumulates within a trial; the maximum trial duration in this protocol (approximately 15 s for the TUG) is short with respect to any realistic clock mismatch between the two devices. The peak-based alignment was preferred precisely because it is independent of the agreement metrics that it feeds.

Cross-correlation and Dynamic Time Warping were considered as automatic alternatives but were deliberately not adopted. Cross-correlation aligns two signals by maximizing their similarity, that is, by optimizing the very quantity that this study sets out to measure, and would therefore have improved the reported agreement by construction. Dynamic Time Warping performs non-linear, locally variable time warping, which would have invalidated the frame-by-frame interpretation of RMSE and concealed any genuine temporal discrepancy.

### 2.4. Statistical Analysis

Statistical analysis was carried out in MATLAB and JMP Pro (version 18.0.2, JMP Statistical Discovery LLC, Cary, NC, USA). Root mean square error (RMSE) and its percentage with respect to the Xsens ROM (%RMSE) quantified the angular error; ROM accuracy was computed as the percentage absolute difference between the ROMs estimated by the two systems. Agreement between the two systems was assessed through Bland–Altman plots, focusing on the bias, defined as the mean difference between the joint angle values estimated by Xsens and those reconstructed from the CapturyStudio keypoints. Reliability was evaluated with the two-way mixed-effects, absolute-agreement, single-rater intraclass correlation coefficient ICC and signal correlation with Spearman’s coefficient (ρ), as normality tests indicated non-normal distributions. Two sets of non-parametric Wilcoxon Rank-Sum tests (α = 0.05) were performed. The first set compared, for each joint angle, the ROM estimated by the two systems to detect any significant difference introduced by replacing the comparison system with CapturyStudio. The second set compared RMSE values to verify the internal coherence of the markerless system, where the absence of a significant difference is the desirable outcome. A side-consistency analysis (left vs. right) was carried out for each joint (EFE left vs. right, SFE left vs. right, KFE left vs. right and HFE left vs. right), and a distal–proximal analysis was performed to assess whether tracking performance changed along the limb. For the upper limb, since the side comparison revealed no significant left–right difference in RMSE, elbow and shoulder values were pooled across sides and compared directly (EFE vs. SFE). For the lower limb, where a significant left–right difference was found, the distal–proximal comparison was kept side-specific (KFE vs. HFE on the left and KFE vs. HFE on the right).

No correction for multiple comparisons was applied. The Wilcoxon tests reported in this work do not constitute a family of hypotheses where each comparison addresses a distinct, pre-specified and joint-specific question—whether the excursion of a given angle is equivalent between the two systems and whether the error of the markerless system is consistent between body sides and along the limb—rather than representing repeated attempts to establish a single scientific claim. The family-wise error rate is therefore not the error rate of interest here. More importantly, the direction of the inferential risk is inverted with respect to the conventional case. In a validation study, the outcome favorable to the system under evaluation is the null hypothesis of no difference from the comparison system, so that any procedure which inflates *p*-values makes the markerless system appear more, rather than less, equivalent to the reference. Correcting for multiplicity would therefore relax, not tighten, the criterion against which the system under evaluation is judged, and reporting uncorrected *p*-values is the conservative choice with respect to the conclusions drawn in this work.

It should finally be recalled that these tests occupy a secondary and descriptive role in the analysis. The primary outcomes of this study are the agreement metrics, RMSE, %RMSE, ROM accuracy, ICC, Spearman’s ρ and Bland–Altman bias with limits of agreement, which are unaffected by multiplicity. The Wilcoxon tests serve only to flag systematic patterns that warrant discussion, and no conclusion of this work rests on the significance of any individual test; the two discrepancies with *p*-values closest to the α = 0.05 threshold (the between-system ROM difference for the right knee and the left–right difference in RMSE for the knee, both with *p* > 0.02) are discussed below as marginal findings and are not used to support any conclusion.

The unit of analysis was the single trial: each of the 198 retained acquisitions contributed one value per metric, and the ten trials recorded for each participant were pooled. This choice reflects the nature of the comparison, which is intra-trial and paired by construction, with both systems measuring the same movement simultaneously, so that the variability of interest is trial-to-trial rather than between-subject. The design is fully balanced, with the same number of trials for every participant, so that pooling does not weight any individual differently.

It must nevertheless be acknowledged that trials recorded from the same participant are not statistically independent observations and that the tests performed on pooled trials therefore operate on an effective sample size smaller than the nominal one. The resulting *p*-values are consequently anti-conservative and are reported as descriptive indicators rather than as confirmatory inference; the conclusions of this work rest on the magnitude of the agreement metrics and not on the significance of these tests.

No a priori power analysis was performed. The sample size was determined by the design conventions of concurrent-validity studies, whose primary outcomes are estimates of agreement rather than tests of a difference, and by the sample sizes adopted in the comparable markerless-validation literature, which typically ranges between 8 and 20 healthy participants [21,22,25,31,32]. The repetition of ten trials per task and per participant was adopted to stabilize the trial-level distributions of the agreement metrics, yielding 198 paired observations per joint angle and permitting the central tendency of RMSE, ICC, ρ and bias to be estimated with narrow interquartile ranges.

## 3. Results

**Figure 6:**
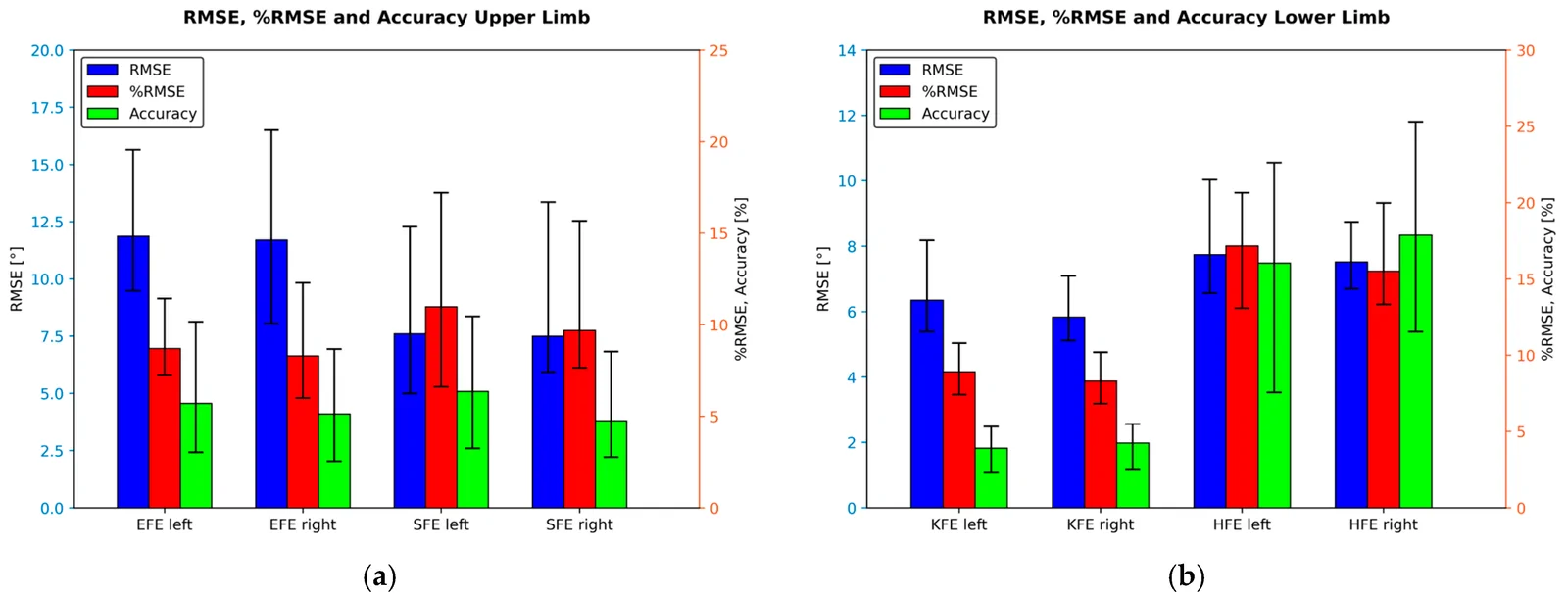
RMSE, %RMSE and ROM accuracy for the upper-limb (**a**) and lower-limb joint angles (**b**).

Statistical indices for all eight joint angles are reported as the median (IQR) in Table 1. RMSE, %RMSE and ROM accuracy are summarized in Figure 6a (upper limb) and Figure 6b (lower limb).

Elbow flexion–extension showed median RMSEs of 11.86° (left) and 11.70° (right); normalizing the error over the joint excursion, the corresponding %RMSE was 8.70% and 8.30%, and the ROM accuracy was 5.70% and 5.13%. Shoulder flexion–extension showed lower absolute errors, with median RMSEs of 7.60° (left) and 7.49° (right), while the smaller angular excursion of the gesture led to a slightly higher %RMSE of 10.98% and 9.68%, with ROM accuracy of 6.35% and 4.76%. The reliability and correlation indices were consistent across sides, with ICCs of 0.95–0.96 for the elbow and 0.94–0.95 for the shoulder, and Spearman’s ρ of 0.98 and 0.97, respectively. The Bland–Altman bias was −8.73° (left) and −9.72° (right) for the elbow, corresponding to an overestimation by CapturyStudio, and 5.12° (left) and 5.28° (right) for the shoulder, corresponding to an underestimation; all bias values remained below 10°, with the differences randomly distributed.

The corresponding indices for the lower limb are reported in Table 1, with the error metrics summarized in Figure 6b. Knee flexion–extension showed median RMSEs of 6.35° (left) and 5.83° (right), with %RMSEs of 8.91% and 8.31% and ROM accuracies of 3.90% and 4.24%. Hip flexion–extension showed median RMSEs of 7.74° (left) and 7.52° (right); here, the reduced angular excursion during walking was reflected in a higher %RMSEs of 17.16% and 15.50% and in a ROM accuracy of 16.04% and 17.87%. ICC was 0.95 for the knee and 0.87–0.88 for the hip, while Spearman’s ρ was 0.89–0.90 for the knee and 0.95 for the hip. The Bland–Altman bias was 1.98° (left) and 1.36° (right) for the knee and 5.22° (left) and 4.86° (right) for the hip, all below 10° and positive, corresponding to an underestimation by CapturyStudio; for the hip, the differences followed a proportional trend, larger for the extension values and approaching zero for the flexion values.

**Figure 7:**
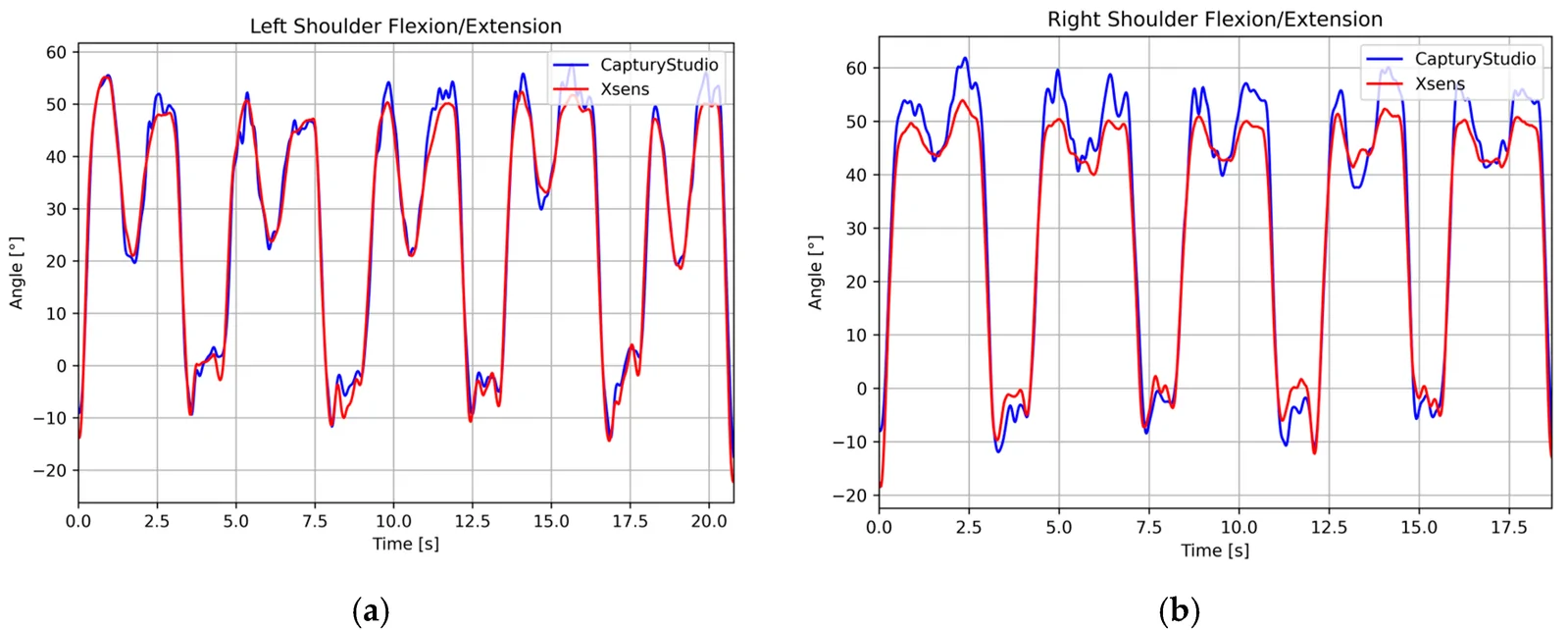

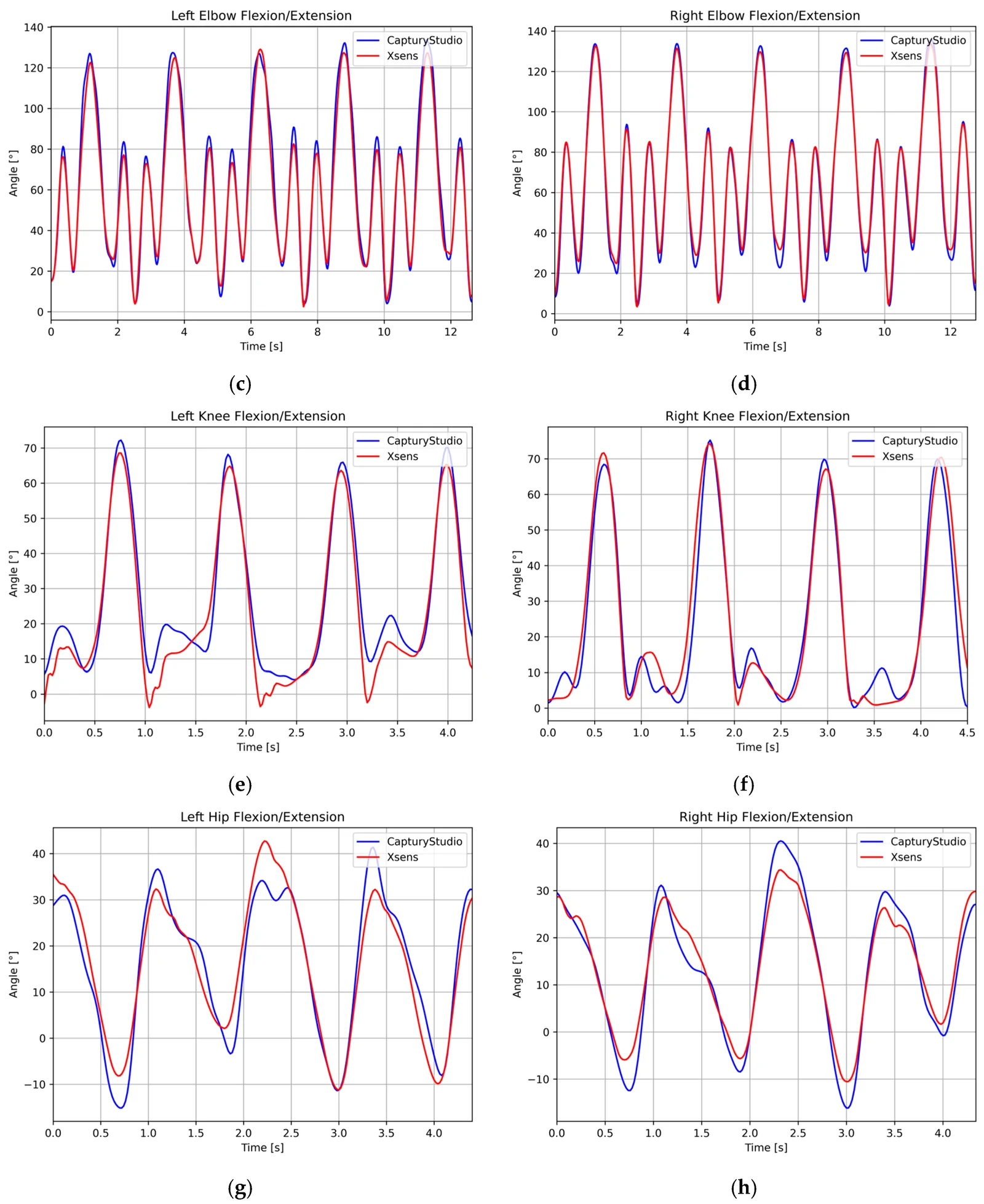
Representative comparison between CapturyStudio (blue) and Xsens (red) angle estimation over time for each analyzed joint angle: left (**a**) and right (**b**) shoulder flexion–extension; left (**c**) and right (**d**) elbow flexion–extension; left (**e**) and right (**f**) knee flexion–extension; left (**g**) and right (**h**) hip flexion–extension.

A representative comparison between the angle signals estimated by the two systems is shown in Figure 7. In all panels, the CapturyStudio signal reproduces the cyclic motor pattern captured by Xsens across the task repetitions. For the shoulder flexion–extension (Figure 7a,b), the angle oscillated between approximately −10° at the extension minima and 50–55° at the flexion peaks, corresponding to an excursion of about 65°, with the two traces closely overlapping. The elbow flexion–extension (Figure 7c,d) spanned from about 5–20° to approximately 125–130°, yielding the widest excursion among the analyzed angles (around 120°). For the knee flexion–extension (Figure 7e,f), the signal ranged from about 0–5° in extension to approximately 70–75° in flexion, an excursion of roughly 70°; the two systems overlapped at the flexion peaks, while the CapturyStudio trace did not reach the same extension minima as Xsens in some cycles. The hip flexion–extension (Figure 7g,h) showed a smaller excursion, oscillating between about −15° in extension and 40° in flexion (around 50–55°); here, the CapturyStudio trace reached more negative values at the extension minima than Xsens, consistent with the ROM difference reported in Table 2.

To assess whether replacing the comparison system altered the estimated joint excursion, the ROM values of the two systems were compared with a Wilcoxon Rank-Sum test for each angle (Table 2). A significant difference emerged only for the left elbow in the upper limb (EFE left *p* = 0.0018), whereas all four lower-limb angles differed significantly (KFE left *p* = 0.0002; KFE right *p* = 0.0276; HFE left and right *p* < 0.0001), with CapturyStudio underestimating the knee ROM and overestimating the hip ROM.

The internal coherence of the markerless system was then examined by comparing RMSE values between body sides and between distal and proximal joints (Table 3). The side comparison showed no significant left–right difference for the elbow (*p* = 0.4174), shoulder (*p* = 0.5792) or hip (*p* = 0.3134) and a significant difference only for the knee (*p* = 0.0439); ROM accuracy showed no significant left–right difference for any joint (EFE *p* = 0.2113; SFE *p* = 0.0677; KFE *p* = 0.4116; HFE *p* = 0.0677). The distal–proximal comparison was significant in both limbs (*p* < 0.0001), with the shoulder error lower than the elbow error in the upper limb and the knee error lower than the hip error on both sides of the lower limb.

## 4. Discussion

The aim of this study was to validate the markerless software CapturyStudio for the reconstruction of upper- and lower-limb flexion–extension kinematics by comparing the angles obtained from its 3D keypoints with those estimated by an Xsens inertial system. Overall, the results are encouraging: the software reconstructed the sagittal-plane angles of elbow, shoulder, knee and hip with errors comparable to those reported in the literature for markerless systems and with reliability and correlation indices that are generally high.

The BTS SMART EVO-DX 2 cameras used here acquire grayscale infrared video, whereas CapturyStudio is optimized for RGB input and explicitly exploits color information to segment and track the subject. The values reported in this study therefore describe the performance of the software under a deliberately unfavorable imaging condition and are best interpreted as a conservative, lower-bound estimate of what the same pipeline can achieve. Users deploying CapturyStudio with RGB hardware should expect equal or better agreement, particularly at the hip, and should not transfer the present figures directly to that configuration; conversely, the fact that the agreement reported below was obtained despite this handicap strengthens rather than weakens the case for the robustness of the underlying tracking.

A first methodological advantage worth noting is that the use of eight cameras surrounding the acquisition volume provides a complete, multi-directional view of the subject, which removes the occlusion problems that a single camera would face (for example, the desk in the RTG task or the change of direction in the TUG test) and allows both limbs to be analyzed within the same session without repositioning the subject. For the elbow, comparable RMSE values were observed between the right and left sides (approximately 12°). This magnitude is smaller than that reported by Scott et al. [33], who obtained an elbow RMSE of 16.3° with limits of agreement from −35.85° to +20.87° using a single-camera markerless set-up during activities of daily living. It should be stressed that those figures are the outcome of a specific preliminary experiment on a single participant and do not constitute a clinically sanctioned acceptability threshold. The comparison therefore indicates that the multi-camera configuration adopted here reduces the error relative to a single-camera one and not that the resulting error is clinically acceptable in absolute terms. The %RMSE values below 10% confirm the quality of the estimate and benefit from the wide angular excursion of the gesture, with ROM frequently exceeding 130°; this is a clear illustration of why the RMSE alone is insufficient and must be normalized to the ROM to be correctly interpreted. The ROM accuracy values also fall within the ranges reported in reaching tasks [34], and both ICC and Spearman’s ρ exceed 0.90, indicating excellent reliability and a strong correlation between the two signals. The Bland–Altman analysis yielded negative biases, indicating a slight overestimation of the joint angle by CapturyStudio; with median values below 10°. This figure should not, however, be presented as a validated acceptability threshold. Choo et al. [35], explicitly indicate that an RMSE up to approximately 5° is what is generally regarded as acceptable for clinical movement analysis and that larger errors may lead to incorrect clinical interpretation; the more permissive 10° figure is discussed in that work in relation to occupational and ergonomic biomechanics, where the required angular resolution is lower, and not as a general medical standard. The bias values obtained here therefore fall within the range considered tolerable for ergonomic and gross movement-pattern applications but exceed the range that would be required for the clinical measurement of absolute joint angles [36]. Inspecting the trials with the highest bias, the RMSE values are comparable to the bias itself, and the Spearman’s coefficients remain very high: this indicates that the error is mainly a systematic offset (an overestimation of the signal), plausibly due to inaccuracies in the reconstruction of the skeleton and of the upper-limb keypoint coordinates rather than to a failure in following the movement. In the best scenarios, the limits of agreement are narrow, while in the worst ones they widen; in all cases, the differences appear randomly distributed, with no discernible proportional pattern. Finally, the Wilcoxon tests showed no significant left–right difference in RMSE, indicating substantially equivalent performance on the two body sides; given the sample size, this should be read as the absence of evidence of a difference rather than as evidence of equivalence. A significant system difference in ROM was found only for the left elbow, but this did not translate into a left–right accuracy difference, confirming the substantial symmetry of performance. During the RTG task, the shoulder is also involved, and its sagittal-plane flexion–extension was analyzed. In line with Bonnechère et al. [32], who highlight that the shoulder is a non-trivial joint to analyze in markerless validation studies, the RMSE values obtained here fell below the 13°, with no statistically significant left–right difference, indicating a consistent detection of the upper-limb keypoints by the software throughout the gesture. Despite the lack of clear thresholds for %RMSE in the literature, the values around 10% can be regarded as encouraging, especially considering that the shoulder excursion in this task is limited; importantly, even for a small ROM, the software was able to estimate the maximum and minimum values, as reflected in the accuracy index. ICC and ρ are again very high, confirming high reliability and strong correlation. The bias values indicate a slight underestimation of the shoulder angle, again within the 10° range, and the limits of agreement are quite narrow in all scenarios, with differences scattered randomly and no systematic trend. The non-parametric tests confirmed that the shoulder performance can be considered equivalent to that of Xsens, with no significant ROM differences on either side and no significant left–right differences in RMSE or accuracy.

Moving to the lower limb, the knee yielded the best overall results among the four joints. The RMSE values remained low with values that are very low if compared to markerless knee estimation [21], and once normalized, the %RMSE fell below the 15% and was comparable between sides. The ROM accuracy was the best of the whole study, below 5% for both knees, while the ICC exceeded 0.90, indicating high reliability and reproducibility. The Spearman’s coefficient, although good, was somewhat lower than for the upper limb, pointing to a strong but not excellent correlation between the waveforms. The bias remained below 5°, and the Bland–Altman limits of agreement were narrow in the best scenarios and slightly wider in the worst, with randomly distributed differences, again indicating equivalence between the two systems. In contrast to the upper limb, the non-parametric tests revealed a left–right difference in RMSE that reached statistical significance (*p* = 0.0439) but was numerically small, with marginally better values for the right knee. Given the proximity of this *p*-value to the α threshold, the number of comparisons performed and the absence of any corresponding difference in ROM accuracy, this finding is regarded as marginal and is not interpreted as evidence of an asymmetry in tracking performance; the side-specific formulation of the distal–proximal comparison was nevertheless retained as the more conservative option, as well as a significant system difference in ROM for both knees, plausibly due to imperfect identification of the leg keypoints and therefore of the extreme angular values. Notably, this RMSE difference did not produce a corresponding accuracy difference between sides, a discrepancy that is explained by the fact that RMSE is computed frame by frame, whereas accuracy depends only on the maximum and minimum values reached.

The hip was the most critical joint of the study. As for the other angles, the absolute RMSE values fall within the band that Bessone et al. [37] classify as tolerable rather than accurate: in that work, an RMSE below 5° is described as acceptable, below 10° as tolerable and above 10° as inaccurate. The hip values obtained here (7.5–7.7°) therefore belong to the intermediate, tolerable category, indicating a reasonable reconstruction of the angular trend. The %RMSE, moreover, rose above the 15% threshold, mainly because of the reduced angular excursion of the hip during walking. The accuracy values were also high, revealing a clear difficulty of CapturyStudio in estimating the hip ROM, which deviated significantly from the Xsens estimate, as confirmed by the Wilcoxon tests on both sides. A closer look at the signals shows that the error is concentrated in the estimation of the minimum angular values (i.e., the hip extension phase during gait) which is systematically underestimated. This negatively affects the reliability of the software for this joint, reflected in ICC values that, although high, are the lowest of the study; nevertheless, the temporal trend is still well captured, as indicated by the high Spearman’s coefficients. Consistently, the bias indicates a slight underestimation, within the 10° limit, and the Bland–Altman plots suggest a proportional pattern, with differences clearly greater than zero for the extension angles and approaching zero for the flexion angles.

The Wilcoxon tests found no left–right differences in RMSE or accuracy for the hip, indicating that no asymmetry in the ROM estimation error was detected. As noted above, however, the present sample supports the detection of substantial asymmetries only, and this finding should not be interpreted as positive evidence of equivalence between sides. Rather, the results suggest a generally symmetric performance of the hip ROM estimation system, even though its overall accuracy remains suboptimal. Taken together, the results indicate a satisfactory performance of CapturyStudio for both limbs, with the RMSE values suggesting a good skeletal reconstruction. The main weakness lies in the hip %RMSE and accuracy, which point to a poorer estimation of the lower-limb ROM; this is consistent with the significant system differences in ROM found for every lower-limb joint and with the findings of Harsted et al. [25], who likewise reported the poorest agreement for the hip when validating CapturyStudio. Two mechanisms may explain this discrepancy, although the present data do not allow their relative contributions to be quantified. First, a definitional difference exists between the reconstructed HFE angle, computed from the thigh vector and a C7-based trunk axis, and the Xsens comparison system, which is defined between pelvis and thigh anatomical frames. Because the reconstructed angle incorporates trunk motion, anterior–posterior trunk lean may be added to the estimated hip motion, likely contributing to the systematic overestimation of hip ROM, particularly during the extension phase. Second, the localization accuracy of the hip and C7 keypoints may also contribute, as both landmarks are inherently more difficult to estimate than the other keypoints used in this study. Since landmark-level ground truth was not available, these mechanisms should be regarded as plausible explanations rather than experimentally verified sources of error. Future work should disentangle their respective contributions. It should also be stressed that while the RTG task is performed seated and engages only the moving limb, the TUG test requires the subject to move through the whole acquisition volume, change walking direction and move the upper and lower limbs simultaneously; all these factors increase the complexity of tracking for the markerless software. A dedicated test comparing the RMSE of proximal and distal joints did not reveal a consistent trend: for the upper limb, the software performed better on the proximal joint (shoulder), whereas for the lower limb, the error was smaller for the distal joint (knee). This suggests that performance depends on the specific joint and on the task rather than on the position of the segment along the limb. Finally, the reliability and correlation indices were highly promising for all angles, although higher for the upper limb, overall demonstrating the ability of the software to recognize the different body segments and to follow their motion over time.

A separate question, and one that the agreement metrics alone do not answer, is whether the magnitude of the discrepancies reported here is compatible with the requirements of clinical measurement. This must be addressed against the measurement error of established clinical practice rather than against generic thresholds. In clinical assessment, the changes in joint angle regarded as meaningful are of the order of a few degrees. Intra-tester goniometric measurement of the elbow and forearm yields minimal detectable changes of approximately 6° in flexion, 7° in extension and 8° in pronation and supination [38]; intra-tester reproducibility of shoulder range of motion in adhesive capsulitis is of the order of 5–7°, although inter-tester differences below 20–25° cannot be distinguished from measurement error [39]; following total knee arthroplasty, a genuine change in knee range of motion must exceed approximately 6° when assessed by the same examiner and approximately 10° when different examiners are involved [40]; and the minimal detectable change for hip internal rotation is approximately 5.4° [41].

The discrepancies measured in the present study, approximately 12° at the elbow, 7.5° at the shoulder and hip and 6° at the knee, with a systematic overestimation of hip ROM of 7.6–9.2°, are therefore of the same order as, or larger than, the changes that would be considered clinically relevant. It follows that the system, in the configuration tested here, cannot be recommended for the measurement of absolute joint angles where a resolution of a few degrees is required: diagnostic goniometry, the certification of range-of-motion deficits and the assessment of small individual changes between visits fall outside its demonstrated capability.

The high ICC and Spearman coefficients establish that the temporal shape of the movement is reproduced faithfully, and this supports a different and narrower class of applications, namely those that depend on the movement pattern rather than on its absolute amplitude: qualitative movement screening, monitoring of gross motor patterns, exercise supervision and real-time feedback in tele-rehabilitation, gamified rehabilitation and group-level or longitudinal analyses in which a systematic offset is common to all measurements and therefore cancels in the comparison. A high correlation between waveforms does not, however, establish the accuracy of the absolute angles, and the two properties must be kept rigorously distinct when the system is proposed for a given application.

Two qualifications apply to this comparison. First, the values reported here are discrepancies with respect to an inertial comparison system that itself carries an uncertainty of 1–5° with respect to stereophotogrammetry, so they constitute an upper bound on the error of the markerless pipeline rather than a direct estimate of it. Second, the clinical figures quoted above refer to manual goniometry, itself a technique with substantial operator dependence; the comparison should therefore be read as a comparison between measurement techniques and not as a verdict on the instrument in isolation.

The analysis was deliberately restricted to sagittal-plane flexion–extension, and this restriction follows from the comparison system rather than from the markerless software. IMU concurrent validity is itself markedly plane-dependent, being high for flexion–extension but appreciably poorer for abduction–adduction and, in particular, for internal–external rotation, where heading error and sensor-to-segment misalignment dominate [8,9,10,11]; a comparison outside the sagittal plane would therefore have quantified the limits of the reference rather than those of the device under evaluation. The two selected tasks are moreover predominantly sagittal, so the available out-of-plane excursions would have been small and the corresponding %RMSE dominated by its denominator. The present results consequently cannot be extrapolated to the frontal and transverse planes, where larger errors are to be expected: markerless systems consistently show their poorest agreement outside the sagittal plane, with the greatest degradation for axial rotation [4,21], and Harsted et al. [25] reported RMSE values up to 16.9° for this same software, with the highest variability precisely in the non-sagittal planes. The conclusions of this study are therefore to be understood as applying to sagittal-plane flexion–extension only; multi-planar validation remains necessary and requires an optoelectronic reference.

A further boundary on the transferability of these results concerns the acquisition environment. The data were collected with eight calibrated cameras, under controlled and constant illumination, with a uniform background, an unobstructed capture volume, standardized movements and healthy young participants, and the reconstruction was performed offline, conditions uniformly more favorable than those of the scenarios in which markerless capture is expected to deliver its principal advantage. In domestic tele-rehabilitation, the viewpoints are typically reduced to one or two, illumination varies, backgrounds are cluttered, furniture occlusions are frequent, and camera placement is not calibrated by an operator; in sporting contexts, velocities are higher, clothing and equipment occlude landmarks, and the capture volume is considerably larger. The reduction in the number of viewpoints is the most critical of these, since the redundancy afforded by eight surrounding cameras is the principal reason why occlusions had limited impact here. The values reported should therefore be regarded as a best-case bound for this pipeline, requiring dedicated re-validation before they are assumed to hold in any reduced-camera or uncontrolled configuration. This has consequences beyond measurement accuracy: in integrated architectures for biomechanical monitoring and adaptive movement control, the kinematic reconstruction is not a product but the input to a control or assistance loop, so that its uncertainty propagates directly into the behavior of the assistive system and into the quality of the feedback delivered to the user [42]. Delimiting the accuracy of the markerless stage, as done here, is thus a precondition for integration into such closed-loop systems.

Several limitations should be considered when interpreting these results, the first of which is instrumental. The BTS SMART EVO-DX 2 cameras acquire grayscale video, whereas CapturyStudio is optimized for RGB footage and explicitly exploits color information to track the subject; the use of monochromatic cameras therefore likely penalized keypoint identification, especially at the hip, and, in a few cases, prevented the software from associating a skeletal model with the subject at all. The sampling frequency, on the other hand, met the software requirements (50 Hz or higher is considered optimal). A second source of limitation concerns the fact that the sample was relatively small and limited to healthy subjects without neurological or musculoskeletal impairments; although ten sessions per task were recorded for each subject to obtain a sufficient dataset for the statistical analysis, the generalizability of the findings remains limited. In particular, the study was not designed to detect between-subject effects: ten participants confer limited power for any such comparison, and the non-significant findings reported above, notably the absence of a left–right difference in RMSE for the elbow, shoulder and hip, must be interpreted as absence of evidence of a difference rather than as evidence of equivalence. Equally, no property requiring repeated assessment over time was investigated: test–retest repeatability across separate visits, responsiveness to a change in clinical status and diagnostic accuracy were not evaluated, and the present design, based on a single acquisition session per participant, does not permit their estimation. It would be especially valuable to investigate the performance of the software on pathological subjects, whose movements tend to be less fluid and more variable, since this is precisely the population for which the system would be used in clinical and rehabilitation settings. Finally, the analysis was restricted to flexion–extension angles in the sagittal plane: extending it to abduction–adduction and internal–external rotation and to additional joints such as the ankle and the wrist as well as trunk bending would provide a more complete picture of the system’s capabilities, which is particularly relevant for clinical rehabilitation assessment. These limitations directly suggest the future developments of this work. The replacement of the grayscale cameras with RGB cameras is expected to improve keypoint recognition, particularly at the hip, and to increase the accuracy of the kinematic estimate even for movements with a small range of motion. Enlarging and diversifying the sample, including individuals with pathologies, would allow a more comprehensive evaluation of the system, while the assessment of additional joint angles and of more complex movements would broaden the scope of validation. A further promising development concerns real-time operation: in this study, the angular reconstruction was performed in post-processing, but implementing the reconstruction algorithm directly within the software would enable real-time feedback of the joint kinematics of the acquired subject, which is a key requirement for interactive rehabilitation and tele-rehabilitation applications.

In conclusion, this study provides a technical characterization of CapturyStudio against an inertial comparison system for sagittal-plane kinematics of the upper and lower limbs. The software reconstructed the temporal pattern of all four joint angles faithfully, with ICC above 0.87 and Spearman coefficients above 0.89 for every angle and with the best agreement obtained for the upper limb. In absolute terms, the discrepancies with respect to the Xsens estimates were approximately 12° at the elbow, 7.5° at the shoulder and hip and 6° at the knee, and the estimation of hip range of motion, systematically overestimated by 7.6–9.2°, emerged as the principal weakness, consistently with the only previous independent validation of this software [25]. These magnitudes are of the same order as the changes regarded as clinically meaningful in goniometric assessment, and they are obtained against a reference that is itself an indirect technique. They therefore support the use of the system for the analysis of movement patterns and for applications tolerant to a systematic offset but not, in the configuration tested here, for the measurement of absolute joint angles in a diagnostic context.

The present work is accordingly to be read as a technical comparison of two measurement systems, performed on healthy young adults in a controlled laboratory environment, and not as a clinical validation. Diagnostic accuracy, between-visit repeatability, responsiveness to a change in clinical status and performance on pathological movement patterns were not assessed, and no conclusion regarding these properties can be drawn from the present data. The clinical, tele-rehabilitative and sporting applications that motivate the development of markerless capture, and towards which the choice of the RTG and TUG tasks is oriented, therefore remain a prospect that this study helps to delimit rather than an established result. Its contribution is to identify where the current accuracy of this pipeline is sufficient and where it is not and to specify the developments required before such applications can be supported by evidence: acquisition with RGB rather than grayscale hardware, a pelvis-fixed definition of the hip angle, validation in the frontal and transverse planes against an optoelectronic reference, validation in reduced-camera and uncontrolled-environment configurations and evaluation on patient populations with larger and more diverse samples.

## Figures and Tables

**Table 1:** Statistical indices comparing CapturyStudio and Xsens for the upper- and lower-limb angles, reported as the median (IQR). EFE: elbow; SFE: shoulder; KFE: knee; HFE: hip flexion–extension. Values are computed over n = 99 trials for the upper-limb angles and n = 99 trials for the lower-limb angles, obtained from 10 participants (10 trials per task per participant; 2 of the 200 acquisitions were excluded owing to errors in skeleton association). ICC values are reported as the median (IQR) across trials.

Angle	RMSE [°]	%RMSE [%]	Accuracy [%]	ICC	ρ	Bias [°]
EFE left	11.86 (6.24)	8.70 (4.22)	5.70 (7.25)	0.95 (0.04)	0.98 (0.01)	−8.73 (8.23)
EFE right	11.70 (8.44)	8.30 (6.27)	5.13 (6.21)	0.96 (0.07)	0.98 (0.01)	−9.72 (12.56)
SFE left	7.60 (7.53)	10.98 (10.60)	6.35 (7.25)	0.94 (0.10)	0.97 (0.02)	5.12 (12.84)
SFE right	7.49 (7.43)	9.68 (8.03)	4.76 (5.83)	0.95 (0.10)	0.97 (0.01)	5.28 (12.88)
KFE left	6.35 (2.79)	8.91 (3.38)	3.90 (2.04)	0.95 (0.04)	0.89 (0.05)	1.98 (3.24)
KFE right	5.83 (1.99)	8.31 (3.37)	4.24 (3.01)	0.95 (0.03)	0.90 (0.07)	1.36 (4.22)
HFE left	7.74 (3.44)	17.16 (7.56)	16.04 (14.92)	0.87 (0.10)	0.95 (0.04)	5.22 (5.24)
HFE right	7.52 (2.13)	15.50 (6.65)	17.87 (15.41)	0.88 (0.07)	0.95 (0.02)	4.86 (4.32)

**Table 2:** Wilcoxon Rank-Sum test on the ROM estimated by the two systems (median (IQR)). * *p* < 0.05 (uncorrected for multiple comparisons).

Angle	ROM Xsens [°]	ROM Captury [°]	*p*-Value
EFE left	135.56 (16.27)	130.49 (11.01)	0.0018 *
EFE right	133.38 (13.85)	131.39 (9.71)	0.3289
SFE left	75.93 (12.46)	73.44 (9.39)	0.8401
SFE right	78.23 (12.03)	77.71 (7.79)	0.6652
KFE left	72.60 (4.75)	70.57 (5.23)	0.0002 *
KFE right	72.15 (5.73)	70.22 (6.42)	0.0276 *
HFE left	48.04 (4.48)	55.61 (4.53)	<0.0001 *
HFE right	47.43 (5.51)	56.63 (5.94)	<0.0001 *

**Table 3:** Wilcoxon Rank-Sum tests on RMSE for left–right and distal–proximal comparisons (median (IQR)), computed over n = 99 trials per limb from 10 participants, as in Table 1. * *p* < 0.05 (uncorrected for multiple comparisons). A and B refer to the first and second condition of each comparison, respectively: for the left–right comparisons, A is the left side and B the right side; for the distal–proximal comparisons, A is the distal joint and B the proximal joint.

Comparison	RMSE A [°]	RMSE B [°]	*p*-Value
EFE left vs. right	11.86 (6.24)	11.70 (8.44)	0.4174
SFE left vs. right	7.60 (7.53)	7.49 (7.43)	0.5792
KFE left vs. right	6.35 (2.79)	5.83 (1.99)	0.0439 *
HFE left vs. right	7.74 (3.44)	7.52 (2.13)	0.3134
EFE vs. SFE	11.85 (7.16)	7.60 (7.78)	<0.0001 *
KFE vs. HFE left	6.35 (2.79)	7.74 (3.44)	<0.0001 *
KFE vs. HFE right	5.83 (1.99)	7.52 (2.13)	<0.0001 *

## Data Availability

Data available on request due to restrictions, e.g., privacy or ethical.
